# The Use of Social Media and Digital Devices Among Italian Neurologists

**DOI:** 10.3389/fneur.2020.00583

**Published:** 2020-06-16

**Authors:** Luigi Lavorgna, Francesco Brigo, Gianmarco Abbadessa, Sebastiano Bucello, Marinella Clerico, Eleonora Cocco, Rosa Iodice, Roberta Lanzillo, Letizia Leocani, Alberto Lerario, Marcello Moccia, Alessandro Padovani, Luca Prosperini, Anna Repice, Maria Stromillo, Francesca Trojsi, Gianluigi Mancardi, Gioacchino Tedeschi, Simona Bonavita

**Affiliations:** ^1^First Division of Neurology, Department of Advanced Medical and Surgical Sciences, AOU – University of Campania “Luigi Vanvitelli”, Naples, Italy; ^2^Division of Neurology, Franz Tappeiner Hospital, Merano, Italy; ^3^Department of Neuroscience, Biomedicine and Movement Science, University of Verona, Verona, Italy; ^4^Second Division of Neurology, Department of Advanced Medical and Surgical Sciences, MRI Research Center SUN-FISM, AOU – University of Campania “Luigi Vanvitelli”, Naples, Italy; ^5^UOSD Neurologia – PO Muscatello di Augusta, ASP Siracusa, Siracusa, Italy; ^6^Clinical and Biological Sciences Department, University of Turin, Turin, Italy; ^7^Department of Medical Sciences and Public Health, University of Cagliari, Cagliari, Italy; ^8^Department of Neuroscience, Reproductive Science and Odontostomatology, Multiple Sclerosis Clinical Care and Research Centre, Federico II University, Naples, Italy; ^9^Neurorehabilitation Unit and INSPE-Institute of Experimental Neurology, Milan, Italy; ^10^Experimental Neurophysiology Unit, Division of Neuroscience, Institute of Experimental Neurology (INSPE), University Vita-Salute San Raffaele, Milan, Italy; ^11^Centro Medico Santagostino, Milan, Italy; ^12^Department of Neuroinflammation, Faculty of Brain Sciences, Queen Square MS Centre, UCL Queen Square Institute of Neurology, University College London, London, United Kingdom; ^13^Neurology Unit, Department of Clinical and Experimental Sciences, University of Brescia, Brescia, Italy; ^14^Department of Neurosciences, S. Camillo-Forlanini Hospital, Circonvallazione Gianicolense, Rome, Italy; ^15^MS Centre SOD Neurologia II. AOU Careggi Largo Brambilla 2, Florence, Italy; ^16^Department of Medicine, Surgery and Neuroscience, University of Siena, Siena, Italy; ^17^First Division of Neurology, Department of Advanced Medical and Surgical Sciences, MRI Research Center SUN-FISM, AOU – University of Campania “Luigi Vanvitelli”, Naples, Italy; ^18^Department of Neuroscience, Rehabilitation, Ophthalmology, Genetics, Maternal and Child Health and CEBR, University of Genova, Genova, Italy

**Keywords:** digital health, social media, digital devices, app, wearable devices

## Abstract

**Background:** Digital devices and online social networks are changing clinical practice. In this study, we explored attitudes, awareness, opinions, and experiences of neurologists toward social media and digital devices.

**Methods:** Each member of the Italian Society of Neurology (SIN) participated in an online survey (January to May 2018) to collect information on their attitude toward digital health.

**Results:** Four hundred and five neurologists participated in the study. At work, 95% of responders use the personal computer, 87% the smartphone, and 43.5% the tablet. These devices are used to obtain health information (91%), maintain contact with colleagues (71%), provide clinical information (59%), and receive updates (67%). Most participants (56%) use social media to communicate with patients, although 65% are against a friendship with them on social media. Most participants interact with patients on social media outside working hours (65.2%) and think that social media have improved (38.0%) or greatly improved (25.4%) the relationship with patients. Most responders (66.7%) have no wearable devices available in clinical practice.

**Conclusion:** Italian neurologists have different practices and views regarding the doctor–patient relationship in social media. The availability of digital devices in daily practice is limited. The use of social networks and digital devices will increasingly permeate into everyday life, bringing a new dimension to health care. The danger is that advancement will not go hand in hand with a legal and cultural adaptation, thus creating ambiguity and risks for clinicians and patients. Neurologists will need to be able to face the opportunities and challenges of this new scenario.

## Introduction

The use of digital devices and the introduction of online social networks have transformed many aspects of clinical practice. Both patients and physicians are increasingly using the Internet and social media platforms to obtain, provide, share, and comment on health information (1, 2). On social media, users can create and share content and can take part in social networking collaborative projects (e.g., Wikipedia), content communities (e.g., YouTube), social networks (e.g., Facebook), web logs (blogs), or virtual games (3, 4). Each of these activities can be used by physicians or by patients to communicate, retrieve, or convey information on health issues or diseases, with an increasing accessibility and widening access, compared to conventional media (5).

An ever-growing number of physicians use social media to share health-related information on a range of conditions, to enhance professional development, but also to facilitate or reinforce doctor–patient relationship, sometimes even providing online consultations (4). This led to some ethical and legal issues, mainly related to the maintenance of boundaries or to the respect of privacy and personal data (6). Finally, digital devices, including wearable devices and exergames (i.e., the use of commercial video games for retraining impaired functions), are increasingly entering the clinical practice, complementing the more traditional tools for monitoring performance or providing exercise (2, 7–11).

So far, few studies have explored attitudes, awareness, opinions, and experiences of neurologists toward social media and digital devices. Thus, we have investigated this in a sample of Italian neurologists.

## Methods

This cross-sectional study was conceived by the study group on “Digital Technology, Web and Social Media” of the SIN (Italian Society of Neurology). Between January and May 2018, each member of the SIN received an e-mail invitation to take part in a written survey aiming to collect information on the attitude of Italian neurologists toward social media and digital devices in the clinical setting. Procedures for obtaining informed consent and protecting participants were approved and monitored by the study group of the SIN coordinating the survey. After having flagged the consent to proceed anonymously (GDPR EU2016/679), the involved neurologist had to fill in a structured survey. A preliminary version of the survey was derived from (4) and circulated among a number of coauthors of this manuscript for internal revision before submission to all participants. The survey mainly consisted of questions aimed at collecting demographic data of responders (age, geographical region), type of digital devices (including wearable devices) available or used in clinical practice and reasons for use, attitude toward social media in communication with patients, and apps used for medical purposes.

Frequencies and percentages were used for the presentation of categorical variables and responses. Three univariable logistic analyses were performed to evaluate the impact of age, sex, and geographical area (recorded in three classes: North, Center, South, and Islands) on the use of social media to communicate with patients. Variables with association with the outcome (*p* <0.01) at the univariable level were then included in a multivariable model. All analyses were performed with Stata 14.1 and *p* <0.05 (two-sided) were considered statistically significant. The study was approved by the Ethical Committee of the University of Campania “Luigi Vanvitelli.”

## Results

A total of 2,434 invitations were sent by e-mails to all members of the SIN. At deadline (May 31), 405 (16.6%) neurologists took part in the study. This sample size gives a margin of alpha error <0.05 considering a confidence level of 95%.

Most participants were aged between 30 and 49 years (50%), 51% (206 out of 405) females and 49% (199 out of 405) males; 31% of the responders were from South Italy, 25% from North-West and 16% from Nord East Italian regions, 18% from Central Italy, and 9% from Italian islands. Most neurologists reported that they were available to their patients irrespective of visiting hours.

At work, 95% of responders use the computer, 87% the smartphone, and 43.5% the tablet. These devices are used to obtain health information (91%), to maintain contact within the medical community (71%), to provide information to colleagues and patients (59%), and to receive clinical updates (67%). Most participants (56%) use social media to communicate with patients, whereas 65% are not in favor of a friendship with patients on social media. The most frequently used social medium at work is WhatsApp (82.5%), followed by Skype (43.6%), Facebook (31.9%), and LinkedIn (29.1%). Similarly, at home, the most used social media are WhatsApp (94.8%), followed by Facebook (65.7%), and Skype (48.9%). Most participants interact with patients on social media outside working hours (65.2%) and think that social media have improved (38.0%) or greatly improved (25.4%) the relationship with patients. Apart from social media, 35% of responders have a personal webpage. In the multivariate analysis, age groups 40–49, 50–59, and 60–69 years and originating from Center and South Italy were associated with higher use of social media to communicate with patients compared, respectively, with age between 20 and 29 years and originating from the North area.

The vast majority of participants (95%) report to have visited patients who had already made a self-diagnosis on the Internet; 70.6% warn their patients against websites providing unreliable or imprecise information, whereas 55.3% advise reliable online sources of information, trying to gain the trust of patients by keeping up to date on health-related news circulating on the Web, demonstrating their unreliability relying on results of scientific studies. Most responders (66.7%) report that they have no wearable devices (i.e., iGloves, eye-trackers, skin patches, or fit watches) available in their clinical practice. The use of consoles like Xbox, Wii, or PlayStation for physical exercise are suggested by 60% of respondents (243 out of 405).

Detailed results and the survey (English version) are provided as Supplementary Material.

## Discussion

To the best of our knowledge, no study has explored how neurologists use social media and digital devices to interact with patients and provide information on diseases yet, though some previous studies assessed the use of social media or digital devices by health care professionals (7).

The most frequent reasons for using social media were to obtain health information, maintain contact within the medical community, provide information to colleagues and patients, and receive clinical updates. These findings emphasize the wide range of opportunities provided to physicians by social media and are consistent with the results of a systematic review that identified the following seven main uses of social media platforms for health communication: (1) providing health information on a range of conditions; (2) providing answers to medical questions; (3) facilitating dialogue between patients to patients, and patients and health professionals; (4) collecting data on patient experiences and opinions; (5) use for health intervention, health promotion, and health education; (6) reducing stigma; and (7) providing online consultations (4).

In our study, about half of the responders (56.3%) reported using social media to communicate with patients; however, 65% of the neurologists were against accepting a friendship with patients on social media. This finding confirms that, despite most participants reporting that social media have improved or greatly improved the relationship with patients, the neurologists' general behavior is aimed at maintaining boundaries in an online doctor–patient relationship. Conversely, the favorable opinion toward friendship with patients expressed by one third of participants raises potential privacy and ethical issues in clinical practice. More specifically, Italian rules on medical confidentiality (Codice di Deontologia Medica, FEDERAZIONE NAZIONALE DEGLI ORDINI DEI MEDICI CHIRURGHI E DEGLI ODONTOIATRI, 2014, updated 2016; available online at: https://www.omceo-to.it/00666/DOCS/8_y-codice-deontologia-medica-2014.pdf) still do not explicitly include or report any specific guidance on securing and sharing patient information on social media or, more generally, on personal online communication.

Online relationship between physicians and patients is indeed viewed by doctors as ethically problematic. Here, the major issues that can arise in online interactions involve the difficulties in setting boundaries or in developing empathy in the doctor–patient relationship due to the lack of physical contact, as well as the therapeutic interaction. These ethics issues are likely to be even more relevant in relationships occurring in social media or social networks. However, the use of social media can prove useful and beneficial to patients through providing and sharing health-related information; it may strengthen professional connections and advance understanding of which individual factors can influence public health (6).

A study conducted among US medical students and physicians showed that most responders considered it not ethically acceptable to interact with patients using online social media and networks, for either social (68.3%) or patient-care (68.0%) reasons (12). Interestingly, 48.7% of responders did not believe that social media could improve patient–doctor communication, also because of problems related to protection of patient confidentiality (79%). However, this study was conducted almost 10 years ago, and the attitude has possibly changed in more recent years. More recently, a survey conducted on 187 Australian doctors showed reluctance to engage with the social media despite the fact that they represent a common feature of clinical practice (13). Although most of them used social media privately, only about 20% had received a “friend request” from a patient. Open issues remained and were specifically related to protection of personal information online and to legal issues (13).

The role of social media to convey health-related information has been evaluated in a few studies. A small survey conducted in 17 physicians emphasized challenges and difficulties arising with this type of communication, including “uncertainty about boundaries or strategies for social media use,” lack of interaction, and the feeling that time spent on social media could be an obstacle to patient care (14).

Our study shows that higher use of social media to communicate with patients was associated with older age and origin from Center and South Italy. This might be explained by the fact that physicians aged between 20 and 29 years, hence just graduated or still residents, do not usually have a deeper relationship with their patients; furthermore, physicians in Northern Italy could be more detached with their patients and less prone to use social media to communicate with them. Interestingly, in our study, almost the total of responders (95%) reported to have visited patients who had already made a self-diagnosis on the Internet, underlying the increasing role of the Internet as a source of medical information by the general population. Most responders tried to develop or enhance a critical attitude of their patients toward information retrieved online, by warning patients against websites providing unreliable or imprecise information, or by even advising reliable online sources of information. This has relevant public health implications, suggesting that instead of discouraging the use of the Internet, physicians should educate patients to a more critical use of it. Furthermore, they could also take advantage of the increasing use of the Internet as a source of information, for instance by gaining the trust of patients by keeping up to date on health-related news circulating on the Web or demonstrating their unreliability by referring to results of scientific studies.

Our survey also assessed the use and/or prescription of wearable devices including exergames in daily practice. Most responders (66.7%) reported that they had no wearable devices (i.e., iGloves, eye-trackers, skin patches, or fit watches) available in their clinical practice. Although we did not address this specific issue, it is likely that wearable devices are more accessible and easy to obtain in the research setting compared to the clinical one. Although they are increasingly used in clinical practice, mainly for rehabilitative purposes, so far, no study has investigated the physicians' attitude toward them (2). However, a survey conducted in physiotherapists and elderly subjects showed that the former are aware of the functions and possible applications of exergames, but they do not think that they will have a relevant influence on traditional rehabilitation tools. Conversely, older people have no interest or even information on their function but could be willing to try them for rehabilitation purposes (15).

There have been great efforts to develop electronic health records providing patients with access to their clinical data. Still, access rights are variable across countries and, so far, this possibility has never been fully explored in Italy (16). However, we cannot exclude that, in the future, electronic health records with patient access and more interactive environment could act as a social platform for customized medical information.

A limitation of this study is the conduction in Italy, a country with the lowest use of the Internet for health information seeking in the European Union (17), and easy-to-access social networks could have compensated this difference that, however, would be expected to reduce over time.

Comparing our data to the surveys available in the literature and previously conducted among physicians, we were not able to identify features indicative of a specific attitude or expectation of neurologists toward social media and digital devices. Italian neurologists have different practices and views regarding the doctor–patient relationship in online social media. The availability of digital devices in daily practice is extremely limited.

Soon, the ever-growing use of online social networks and availability of digital devices will increasingly permeate into everyday life, bringing a new dimension to health care. Benefits will include the increased availability to generate, share, and comment on health issues, with the ultimate aim of improving health outcomes and communication practices. However, this also carries risks associated with spreading unreliable or low-quality information and protection of informational privacy. Rules on medical confidentiality should formally address the issue of securing and sharing online patient information as well as the relationship with patients on social media. The greatest danger is that technological advancement will not go hand in hand with a legal and cultural adaptation, thus creating ambiguity and risks for clinicians and patients. Neurologists and health care personnel will need to be able to face the opportunities and challenges of this new scenario.

## Data Availability Statement

All datasets generated for this study are included in the article/Supplementary Material.

## Author Contributions

All co-authors have made a substantial contribution to the design, data collection, analysis of the research, drafting of the manuscript, and have reviewed and accepted the contents of the manuscript prior to its submission.

## Conflict of Interest

The authors declare that the research was conducted in the absence of any commercial or financial relationships that could be construed as a potential conflict of interest. Reviewer KS declared a past co-authorship with one of the authors, LLa, to the handling editor.
