# Supplementary material for: The Use of Social Media and Digital Devices Among Italian Neurologists
Source: Front Neurol. 2020 Jun 16;11:583. doi: 10.3389/fneur.2020.00583 (PMC7308485; doi:10.3389/fneur.2020.00583)
Supplement: Supplementary file 1 [file Data_Sheet_1.PDF]

Supplementary material - Digital and Neurologists

Table 1: population distribution according to age

Age	Distribution of responders N	%
20-29	31	8
30-39	107	26
40-49	98	24
50-59	91	22
60-69	67	17
70-79	5	1
80-90	6	1

Table 1: population distribution according to geographical region

area	N	%
Nord West	101	24.9
Nord Est	65	16.1
Center	74	18.3
South	127	31.4
Islands	38	9.4

Table 2

My advices, my knowledge and my professionalism are available to patients	N	%
By any means, irrespective of visiting hours	284	70.1
Only in the clinic/hospital/medical office I avoid contact with patients outside of my work environment.	121	29.9

Table 3

Which device do you use in your professional environment? -	N	%
Smartphone	352	86.9
Tablet	176	43.5
Personal computer	385	95.1

None	4	1.0
Other	3	0.7

Table 4

What is the main purpose of using your device in clinical practice	N	%
To follow the disease evolution over time	271	66.9
To provide information to colleagues and patients	239	59.0
To keep up to date on health information circulating online	370	91.4
To maintain contact within the medical community	289	71.4

Table 5

		N	%
Communicate with patients using social media		228	56.3
Are you in favor of using social media with patients (1= not at all; 4= very much?			
Not at all		98	24.2
	2	68	16.8
	3	100	24.7
	4	58	14.3
Very much		81	20.0
Are you in favor of friendship with patients in social media (1= not at all; 4= very much)?			
Not at all		200	49.4
	2	62	15.3
	3	46	11.4
	4	25	6.2
Very much		72	17.8

Table 7

Which social media do you use for working purposes at work and at home?

	At work		At home	
	N	%	N	%
None	19	4.7	5	1.2
Whatsapp	334	82.5	384	94.8

Facebook	129	31.9	266	65.7
Twitter	75	18.5	112	27.7
Goolge+	69	17.0	102	25.2
Linkedin	118	29.1	91	22.5
Wikipedia	115	28.4	130	32.1
Youtube	81	20.0	152	37.5
Quora, Reddit, Yahoo Answers or similar	29	7.2	16	4.0
Forum	91	22.5	48	11.9
Skype	140	34.6	198	48.9
Facetime	37	9.1	81	20.0
Other professional social networks	121	29.9		0.0

Table 6

	N	%
I communicate with patients on social media outside the visiting hours	264	65.2
How did social media influence the relationship with patients?		
The relationship has greatly worsened: there is no distance between patients and physicians and I am being consulted for every symptom	25	6.2
The relationship has worsened: I can't keep the professional and private fields separate	43	10.6
The relationship has not changed much compared to the past	80	19.8
The relationship has improved: With due limits, it is a more direct and practical way to communicate with the patient	154	38.0
The relationship has greatly improved: Communication is more immediate and I can follow my patients with greater continuity	103	25.4

Table 7

Besides social media, how are you active as Neurologist in the Internet?	N	%
I am not present online	143	35.3
Personal Website	142	35.1
Blog	79	19.5
Podcast	30	7.4
Forum	62	15.3
YouTube Channel	30	7.4
Dottori.it or similar websites	93	23.0
Other	117	28.9

Table 8

How do you help patients to consult the Web to obtain medical information? -	N	%
I do not provide them any advice	8	2.0
I recommend reliable websites with scientific rigour	224	55.3
I warn patients from websites that provide inaccurate and untrustworthy news	286	70.6
I update the patient on the latest fake news circulating on the Web related to his/her disease	165	40.7
I recommend to register to targeted social networks which are serious and trustworthy	110	27.2

Table 9

The easy availability of medical information on the WEB is one of the reasons why the doctor-patient relationship, first based on trust and acquiescence (compliance), has mutated. How do you try to adjust?	N	%
I try to regain the confidence of the patient by showing me updated on the news that run on the WEB and demonstrating the unreliability of some of these on the basis of scientific studies.	274	67.7
I do not follow much the Internet, but I am willing to explain to the most stubborn patients the reason for my medical opinions.	77	19.0
I prefer not to question therapies and diagnoses with patients. My opinion is the result of study and experience and it must be enough to receive confidence from patients.	54	13.3

Table 10

What is your attitude towards wearable devices?	N	%
I don't know what the wearable devices are	37	9.1
We do not have wearable devices	270	66.7
I am against the use of wearable devices	34	8.4

Which wearable devices are available in your clinic? And which would you desire to have available?

	Available		Desidered	
	N	%	N	%
iGloves	52	12.8	149	36.8
Eye-tracker	47	11.6	175	43.2
Skin patch	52	12.8	170	42.0
Fit watch	73	18.0	170	42.0
Others	7	1.7	136	33.6

Table 11

What medical apps do you recommend to your patients?

	N	%
Booking apps	1	1.5
Brian training nintendo	1	1.5
app for brain training	2	3.0
Fitness, Cognitive rehabilitation	1	1.5
fitness or concentration games	1	1.5
Coordinating games on consoles	2	3.0
App monitoring physical activity	2	3.0
Apps for mental and physical exercises	1	1.5
App for concentration and motor exercises	1	1.5
Fitness and health	1	1.5
Fitness	3	4.5
Fitness, FitBit	1	1.5
Logic games on the phone	1	1.5
Head app	1	1.5
Medscape	1	1.5
My Voice	1	1.5
Nothing	33	49.3
Wikipedia	1	1.5
App to count steps	2	3.0
Headache diary	3	4.5
pubmed	1	1.5
Puzzle games and fitness apps	1	1.5
SM Mobile, Neuro-Compass	1	1.5
sclerosi multipla-e.it	1	1.5
Targeted Social network;	1	1.5

Onederful	1	1.5
salutelazio 112-where are u	1	1.5

Table 12

What apps do you use for medical purposes?

	N	%
Booking apps	1	1.2
App with medication list and description	1	
Anatomy Atlas. Pharmaceutical Prontuario	1	1.2
Steps and Beat Count	1	
Device for Motor rehabilitation	1	
Edss calculator nervewhiz	1	
Epocrates	1	1.2
Farmabank, Qxd Calculate, prontuario ..	1	
Fitbit	4	
I-Med; Smart-Pharma	1	1.2
I-Med; Smart-Pharma; Steroid converter	1	
IFarmaci	1	1.2
IFarmaci, edss calcolatore, Glasgow c..	1	1.2
IVT aspirin guide ASCVDplus medcalcx ..	1	1.2
MEDSCAPE	11	13.4
Medscape, app of scientific journals	1	1.2
Medi math	1	1.2
MediWeb, iPharma	1	1.2
Medscape, Eyehandbook	1	1.2
Medscape iFarmaci iMWHIcd	1	1.2
Medscape, MedCalc, FarmaciaPlus, CUF ..	1	1.2
Medscape, smartpharma	1	1.2
Nothin	16	19.5
NeuroToolKit, MediMath, Medscape	1	1.2
Nihhs gcs	1	1.2
QRead, Farmaci	1	1.2
Onederful	1	1.2
Clinical scales	1	1.2
Skype, whatsapp, facebook	1	1.2
Smart Pharma, medscape	1	1.2
SmartPharma	2	2.4
UPToDate, Stroke Scale	1	1.2
UpToDate	1	1.2
Uptodate, SmartPharma, NIHSS, Neurolo..	1	1.2
Wikipedia	1	1.2
Calculators, medscape, iFarmaci	1	1.2
Score calculation, dosage medications	1	1.2
Headache diary	1	1.2
App to count steps	1	1.2

farmabank medscaps	1	1.2
fitbit	1	1.2
iFarmaci	2	2.4
iFarmaci NIHSS	1	1.2
The same mentioned in the previous question	1	1.2
medcalc neurotoolkit medscape NAO, MD..	1	1.2
Several. Neurotoolkit, Medimath, ifa..	1	1.2
pubmed	1	1.2
pubmed, prontuario	1	1.2
smartpharma	1	1.2
torrinomedica, uptodate	1	1.2
uptodate	1	1.2
uptodate, medscape, smartpharma, sanf..	1	1.2

Do you communicate with patients using social media?

Table 15

		No		Yes		Univariate			Multivariate		
Age		N	%	N	%	OR	CI95%	p	OR	CI95%	p
	20-29	19	61.3	12	38.7	1			1		
	30-39	56	52.3	51	47.7	1.44	0.64-3.26	0.38	1.68	0.71-3.94	0.233
	40-49	31	31.6	67	68.4	3.42	1.48-7.92	0.004	3.91	1.63-9.41	0.002
	50-59	42	46.2	49	53.8	1.85	0.80-4.24	0.148	2.45	1.02-5.87	0.045
	60-69	21	31.3	46	68.7	3.47	1.43-8.43	0.006	3.83	1.51-9.71	0.005
	70+	8	72.7	3	27.3	0.59	0.13-2.69	0.499	0.87	0.18-4.17	0.859
Gender											
	F	91	44.2	115	55.8	1					
	M	86	43.2	113	56.8	1.04	0.70-1.54	0.846			
Geographic area											
	Nord	101	60.8	65	39.2	1			1		
	Center	27	36.5	47	63.5	2.7	1.53-4.77	<0.001	2.41	1.34-4.34	0.003
	South and Islands	49	29.7	116	70.3	3.68	2.33-5.81	<0.001	3.73	2.32-5.98	<0.001

Table 13

Have you visited patients who had already made a self-diagnosis on the Internet?	N	%
	386	95.0
